# Flixweed Is More Competitive than Winter Wheat under Ozone Pollution: Evidences from Membrane Lipid Peroxidation, Antioxidant Enzymes and Biomass

**DOI:** 10.1371/journal.pone.0060109

**Published:** 2013-03-22

**Authors:** Cai-Hong Li, Tian-Zuo Wang, Yong Li, Yan-Hai Zheng, Gao-Ming Jiang

**Affiliations:** 1 State Key Laboratory of Vegetation and Environmental Change, Institute of Botany, the Chinese Academy of Sciences, Beijing, PR China; 2 University of Chinese Academy of Sciences, Beijing, PR China; Ben-Gurion University, Israel

## Abstract

To investigate the effects of ozone on winter wheat and flixweed under competition, two species were exposed to ambient, elevated and high [O_3_] for 30 days, planted singly or in mixculture. Eco-physiological responses were examined at different [O_3_] and fumigating time. Ozone reduced the contents of chlorophyll, increased the accumulation of H_2_O_2_ and malondialdehyde in both wheat and flixweed. The effects of competition on chlorophyll content of wheat emerged at elevated and high [O_3_], while that of flixweed emerged only at high [O_3_]. The increase of H_2_O_2_ and malondialdehyde of flixweed was less than that of wheat under the same condition. Antioxidant enzyme activities of wheat and flixweed were seriously depressed by perennial and serious treatment using O_3_. However, short-term and moderate fumigation increased the activities of SOD and POD of wheat, and CAT of flixweed. The expression levels of antioxidant enzymes related genes provided explanation for these results. Furthermore, the increase of CAT expression of flixweed was much higher than that of SOD and POD expression of wheat. Ozone and competition resulted in significant reductions in biomass and grain yield in both winter wheat and flixweed. However, the negative effects on flixweed were less than wheat. Our results demonstrated that winter wheat is more sensitive to O_3_ and competition than flixweed, providing valuable data for further investigation on responses of winter wheat to ozone pollution, in particular combined with species competition.

## Introduction

Atmosphere ozone (O_3_) at ground level is one of the most serious air pollutants, which negatively affects crop growth and yield [1]. The tropospheric [O_3_] is predicted to rise globally by 40–60% by 2100 as a consequence of rapid economic development [2], suggesting O_3_ pollution under global change scenarios will become more serious. The potential impact of elevated O_3_ on agricultural productivity is particularly relevant in the south and East Asian region. For instance, it is estimated that by 2020 current day, the yield losses due to O_3_ damage will rise from current 13% and 23%, to 16% and 35%, respectively for cereals and soybean productions of China [3], [4]. Economically, annual crop losses induced by O_3_ are estimated at $3–5.5 billion in China, and will likely increase by 16%–110% in the near future [5], [6]. These predictions suggest that China in particular might be on the cusp of substantial reductions in grain production following the rapid economic transformation, industrialization and urbanization [7].

Among major food crops, wheat is believed to be the most sensitive to O_3_ [8]. Such a phenomenon has attracted attentions by a number of scientists from the view of food security [9], [10]. Previous studies have found that O_3_ negatively influence the growth and development of plants [11], [12]. Acute and chronic exposure to O_3_ can pressurize plants to bring a series of changes, such as appearances of visible symptoms, premature senescence, yield losses and alterations of plant community structure [13]. It is well known that ozone affects primarily growth of plant by producing reactive oxygen species (ROS), which severely compromise the integrity of metabolically important membranes [14]. The concept that antioxidant enzymes act as a barrier against toxic oxygen derivatives, as initially suggested in the 1970s, became dominant. Over the 1980s and 1990s, many studies investigated the involvement of antioxidants in plant resistance to different forms of abiotic stresses [15]. Therefore, antioxidant enzymes which have the ability of detoxifying ROS, plays important roles in engendering tolerance to O_3_ in different species [16].

Formerly, most of the researches revealed the response of plants to O_3_ at the level of individual species. There are few investigations on the indirect impact of O_3_ pollution on growth and yield performance under the competition from weed. Flixweed (*Descurainia Sophia*) is the most troublesome annual weed, widely occurred in the major wheat planting regions of China. It is a vigorous competitor with prolific seed production, with the ability to quickly invade wheat fields. More seriously, it has developed strong resistance to herbicides, becoming a great threat to wheat production [17], [18]. The presence of weeds with other crops decreases grain yields owing to competition for light, moisture and minerals [19]. Allelopathic functioning from weeds is another important factor to cause detrimental effect on the growth of the crops by releasing allelochemicals into the ambient environment [20], [21].

From the above mentioned introduction, winter wheat confronts not only O_3_ stress under global climate change scenarios, but also the threat caused by weeds such as flixweed. Li *et al.* (1999) have found that the relative competitive status of wheat competing with wild oat decreased grain production under UV-B enhancement [22]. It has been well documented that competition is an important factor affecting plant responses to ozone stress [23]. Nevertheless, we never know the performing patterns of competition from wheat and weeds under O_3_ pollution. Therefore, it is urgent to assess the interactive effect on winter wheat and flixweed under O_3_ stress and competition.

In the present study, we investigated the effects of O_3_ on winter wheat and flixweed under competition. The objectives of this investigation are: 1) to determine the effects of O_3_ on growth and yield of winter wheat and flixweed; 2) to investigate the physiological mechanism of their responses to O_3_ and competition. This research may provide some valuable information for developing the necessary weed management strategies and theoretical foundation for future wheat breeding.

## Materials and Methods

### Plant culture and O_3_ fumigation

Winter wheat (*T. aestivum*. cv Liangxing99) and flixweed (*Descurainia Sophia*) were selected in the experiment. Flixweed grows as a common and competitive weed occurring in wheat fields of North China, with a growth season being from October to May. On 12 October 2010, 15 seeds of winter wheat or 15 seeds of flixweed were sowed in each pot, with total of 54 plastic pots (25 cm in diameter, 28 cm in height). For the competition experiment, 15 wheat seeds together with 15 seeds of flixweed were planted in each of the 54 plastic pots. Pots were all filled with clay soil containing organic C, total N, available P and K at the rate of 1.3 g/kg, 0.73 g/kg, 67 mg/kg, and 157 mg/kg, respectively. No chemical fertilizers were applied either as basal or topdressing. Monocultures of winter wheat and flixweed were both thinned to ten per pot after their emergence. Mixcultures had ten wheat seedlings and ten flixweed seedlings per pot.

On 1 April 2011, six pots of either monoculture or mixculture were moved to each of nine open-top chambers (OTC, 2.6 m in diameter, 2.4 m in height) built in the open field. Plants were allowed to adapt to chamber environments for 7 days before O_3_ exposure. During this adaptation period, all plants received ambient air with an O_3_ concentration of less than 40 ppb. The gas dispensing system of the OTCs was conducted according to Uprety (1998) [24]. Ozone was added to the open air entering three of the chambers to maintain an O_3_ concentration of 80±5ppb for 7 h day^−1^ (10:00–17:00 hours) for 30 days. Another three chambers were set up the same way but with higher O_3_ concentration of 120±10 ppb. Meanwhile, three chambers were ventilated with ambient air as the control. The injected O_3_ was generated by electrical discharge using ambient air with an O_3_ generator (CF-KG1, Shanmeishuimei Ltd., Beijing, China). The mean daily temperature and the mean monthly precipitation were shown in Figure S1.

During the experimental run, plants were watered every two or three days to avoid water deficit. The O_3_ treatments (high [O_3_] 120 ppb; elevated [O_3_] 80 ppb; ambient [O_3_]<40 ppb) were assigned randomly to the chambers and replicated thrice. To minimize the effect of chamber and environmental heterogeneity which may potentially affect the results, we switched plants and associated O_3_ treatment among chambers and locations of the plants within the chambers and further randomized every five day.

### Plant sampling

After one day of O_3_ fumigation, the most recently expanded leaves on the main stem of wheat and flixweed from differential treatments were collected from each chamber per treatment for biochemical measurements. Then the most recently expanded leaves were sampled every ten days. Leaf samples were immediately frozen in liquid nitrogen then transferred to an ultra-freezer at −80°C until the time of assay.

### Chlorophyll content

Chlorophyll was extracted from fresh leaf samples (0.2 g) in 95% ethanol in the dark for 48 h at 4°C. The extract was then assayed for chlorophyll with the absorption spectra provided by Arnon (1949) [25].

### Determination of H_2_O_2_ and malondialdehyde (MDA)

Hydrogen peroxide (H_2_O_2_) and malondialdehyde (MDA) in leaves were measured to assess the effect of O_3_ on oxidative damage of wheat and flixweed. H_2_O_2_ content was measured according to the method described by Alexieva *et al*. (2001) [26]. MDA was determined according to the method of Kramer *et al*. (1991) [27].

### Determination of the activity of antioxidant enzymes

Leaf samples (0.5 g) were homogenized in a prechilled mortar and pestle placed on ice with 3 mL 0.1 M potassium phosphate buffer (pH 7.8). The homogenate was centrifuged at 1,200 *g* for 20 min at 4°C and the supernatant was used to determine enzymes activities.

Superoxide dismutase (SOD) activity was measured spectrophotometrically based on inhibition in the photochemical reduction of nitroblue tetrazolium described by Giannopolitis and Ries (1977) [28]. One unit of SOD is defined as the amount of enzyme that inhibited the nitroblue tetrazolium reduction by 50%. Catalase (CAT) activity was assayed using the method of Aebi (1984) [29]. Unit of CAT activity was defined as variation of absorbance per minute per gram total protein. Peroxidase (POD) was determined through measuring the oxidation of guaiacol [30]. Total soluble protein was measured by the method of coomassie brilliant blue [31].

### Expression patterns of antioxidant enzyme related genes

The response of genes to environmental change was much quicker than physiological reaction, so the expression of antioxidant enzyme related genes were determined after one day exposure by real-time quantitative PCR (RT-qPCR). Total RNAs were extracted from harvested leaves of wheat and flixweed with Trizol reagent (Invitrogen) after one day fumigating. The total RNAs were reversely transcribed into first-strand cDNA with PrimeScript® RT reagent Kit With gDNA Eraser (TaKaRa), and the cDNAs obtained were used as templates for PCR amplification with specific primers. Given the full genome of flixweed haven't been sequenced, primes of flixweed were designed according to *Arabidopsis thaliana* which is in the same family—*Cruciferae*. Gene-specific primers used for RT-qPCR were: 5′-TTG TAG GTC GCT GGT TTC-3′ and 5′-CCA AGT TCA CGG TTC ATA G-3′ for *TaSOD* (U69536.1); 5′-AGT TGG ACG GAT GGT ACT GA-3′ and 5′-AAG ACG GTG CCT TTG GGT-3′ for *TaCAT* (X94352.1); 5′-GAC GGC TGA ATG GTT GAA-3′ and 5′-AAT GCC TCC TGG TCC TCT-3′ for *TaAPX* (AF387739.1); 5′-AAC TAC CCG CTC TGC TCC T-3′ and 5′-GCC TTG GTC CTT GTA CTT CG-3′ for *TaGPX* (AJ010455.1); 5′-TGA ACA GCA GTG AGG GTG-3′ and 5′- GAG TTT GGT CCA GTA AGA GG-3′ for *DsSOD* (NM_100757.3); 5′-CTC TTC CCT CAC CAT CGG-3′ and 5′-TGG AGA AAC GGA CAA TAA CC-3′ for *DsCAT* (NM_001035995.3); 5′-GGT CGG ATG GGA CTC AAT-3′ and 5′-AGA GCC TTG TCG GTT GGT-3′ for *DsAPX* (NM_111798.3); 5′-GGT GGA TGT GAA CGG TAA G-3′ and 5′-CCA ACG CAG TTT GAA TGT C-3′ for *DsGPX* (NM_128714.3). In addition, *Actin* was used as internal control: 5′-CTA TCC TTC GTT TGG ACC TT-3′ and 5′-AGC GAG CTT CTC CTT TAT GT-3′ for *TaActin* (AB181991.1); 5′-TGT TCT TTC CCT CTA CGC T-3′ and 5′-CCT TAC GAT TTC ACG CTC T-3′ for *DsActin* (NM_112046.3). RT-qPCR was performed using Stratagene Mx3000P^™^ instrument. Each reaction contained 7.5 µl 2×SYBR Green Master Mix reagent (TaKaRa), 0.5 µl cDNA samples, 0.6 µl 10 mM gene-specific primers and 0.3 µl 50×ROX in a final volume of 15 µl. The thermal cycle was used as followings: 95°C for 2 min; 40 cycles of 95°C for 30s, 55°C for 30s, and 72°C for 30s. The relative expression level was analyzed by the comparative Ct method and the value of AS was normalized to one.

### Biomass and grain yield

Five plants per pot for each species and each treatment (n = 15) were harvested to determine the biomass and grain yield. Plants were dried to constant weight in an oven at 72°C. Grains were removed from each ear or pod by hand. Yield per plant was determined for sundried seeds.

### Statistical analysis

The experiment was arranged as a split plot with three replications. O_3_ concentration levels (ambient [O_3_]<40ppb, elevated [O_3_] 80±5ppb, high [O_3_] 120±10 ppb) represent the main difference. Wheat and flixweed population ratio (10∶0; 10∶10; 0∶10) was the subplot. One-way ANOVA of SPSS package (Ver. 17, SPSS, Chicago, USA) with Tukey-Kramer multiple comparison tests were performed to test for significant differences between the control and other treatments. Differences among treatments were considered significant if *P*≤0.05.

## Results

### Chlorophyll content

**Figure 1:**
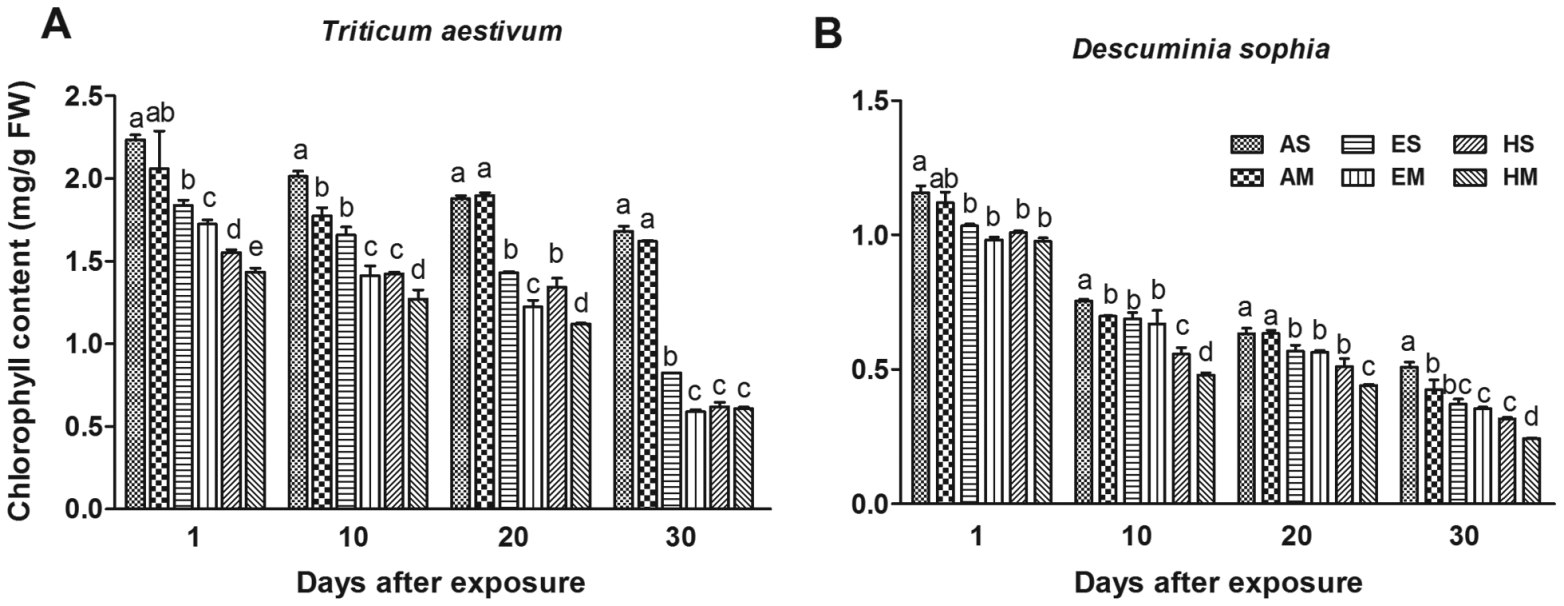
Effects of different ozone concentrations on chlorophyll contents. Contents of chlorophyll in leaves of winter wheat and flixweed were shown in panel A and B, respectively. AS and AM represent winter wheat or flixweed grown singly and in mixture in ambient air, respectively. ES and EM represent winter wheat or flixweed grown singly and in mixture in elevated O_3_ concentration, respectively. HS and HM are winter wheat or flixweed planted singly and in mixture in high O_3_ concentration, respectively. Error bar indicates SE. *n* = 9. T bars with different letters are significantly different between treatments at *P*≤0.05 at the same sampling time.

As shown in Figure 1A, at the absence or presence of flixweed, the chlorophyll contents of wheat decreased in varying degrees compared with those growing singly in ambient air (AS). On the second day of O_3_ exposure, the contents of chlorophyll were significantly decreased by 18%, 23%, 31% and 36% for wheat planted singly in elevated [O_3_] (ES), planted in mixture in elevated [O_3_] (EM), planted singly in high [O_3_] (HS) and planted in mixture in high [O_3_] (HM) against AS, respectively (*P*≤0.01, Figure 1A). However, there were no significant differences between those planted in mixture in ambient air (AM) and AS treatments. Along with the exposure time, the adverse effect induced by O_3_ was enforced gradually.

The present of O_3_ also reduced the chlorophyll contents of flixweed. At the end of exposure, elevated [O_3_] (80 ppb) significantly reduced the chlorophyll content of flixweed by 27% and 31%, respectively for ES and EM treatments against AS (*P*≤0.05, Figure 1B). While in the case of the high [O_3_] (120 ppb), the chlorophyll content was dramatically being 38% and 52% lower in HS and HM than that of AS (*P*≤0.01, Figure 1B).

The adverse effects of O_3_ on chlorophyll content were obvious for both of winter wheat and flixweed, as well as competition exsited in mixculture treatments. However, the effects of competition on chlorophyll content of wheat emerged at elevated and high [O_3_], while that of flixweed emerged only at high [O_3_].

### H_2_O_2_ and MDA contents

**Figure 2:**
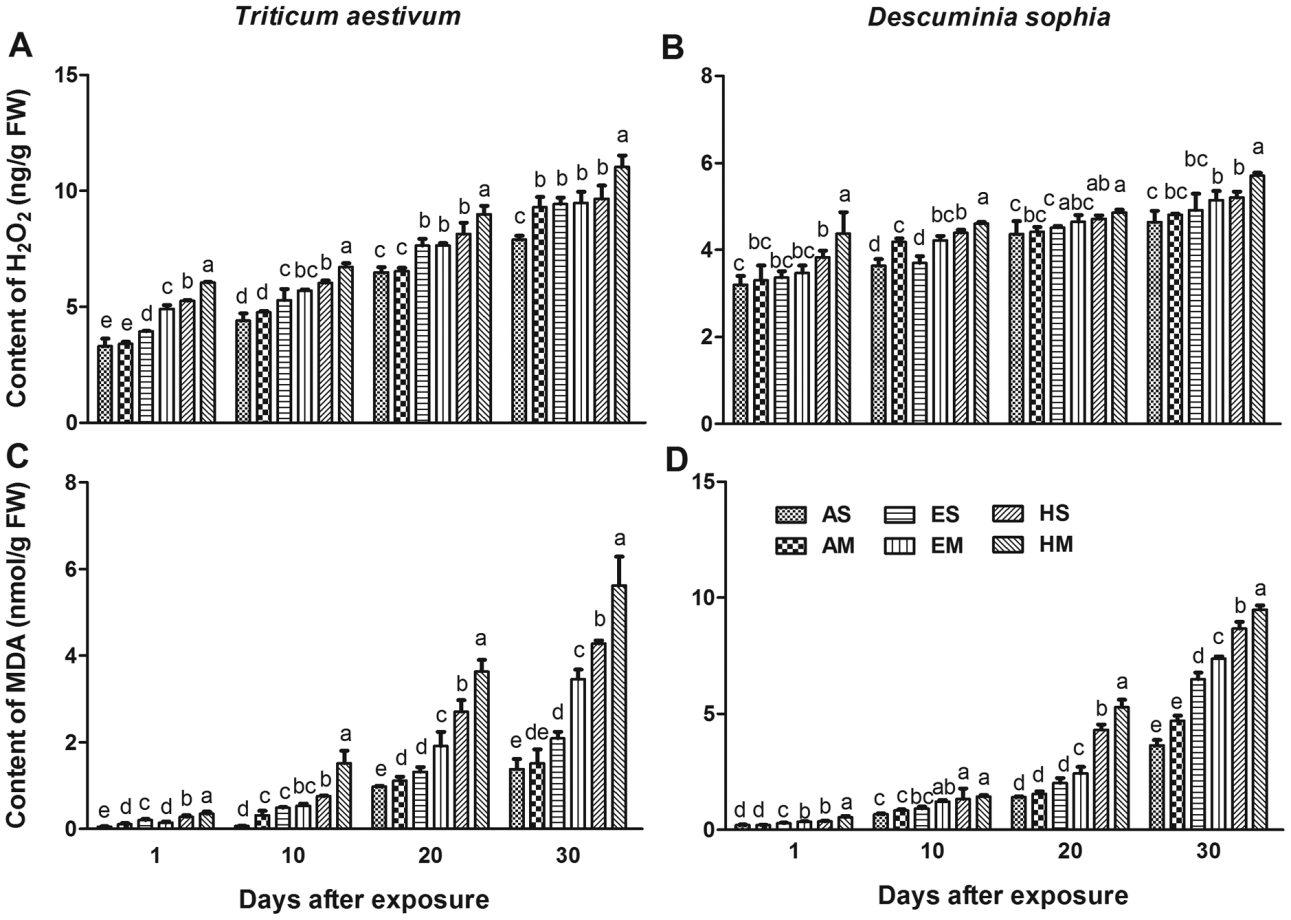
Effects of ozone on hydrogen peroxide and malondialdehyde contents. H_2_O_2_ contents in leaves of wheat and flixweed were shown in panel A and B, respectively. Malondialdehyde contents in leaves of wheat and flixweed were shown in panel C and D, respectively. Columns with error bars are mean±SE of six replicates. T bars with different letters are significantly different between treatments at *P*≤0.05 at the same sampling time.

Hydrogen peroxide (H_2_O_2_) is one kind of ROS induced by adverse stress. Ozone significantly increased H_2_O_2_ content both in winter wheat and flixweed leaves (Figure 2A, B). For winter wheat, at the end of exposure period, the H_2_O_2_ contents were notably increased by 17%, 19%, 20%, 22% and 40%, respectively for AM, ES, EM, HS and HM against AS (Figure 2A). Nevertheless, in absence or presence of wheat, the increase of H_2_O_2_ contents from flixweed leaves was only found to be significant in EM (10%), HS (11%) and HM (21%) treatments against AS (*P*≤0.05, Figure 2B). Meanwhile, species competition between wheat and flixweed growing in mixculture, significantly enhanced the content of H_2_O_2_ for both wheat and flixweed at high [O_3_] (*P*≤0.05).

Increase of malondialdehyde (MDA) content, which has been widely recognized as a parameter for lipid peroxidation, was observed in wheat plants exposed to O_3_ pollution (Figure 2C). Compared with the AS treatment, O_3_ significantly enhanced MDA contents by approximately 50%, 150%, 200%, and 300% respectively for ES, EM, HS and HM at the end of exposure. Similar change tendency of MDA content was detected in flixweed plants. MDA contents were notably increased by 78%, 102%, 138% and 160% for ES, EM, HS and HM relative to AS in flixweed (Figure 2D). However, the increase of flixweed was less than that of wheat under the same condition.

### Antioxidant enzymes activity

**Figure 3:**
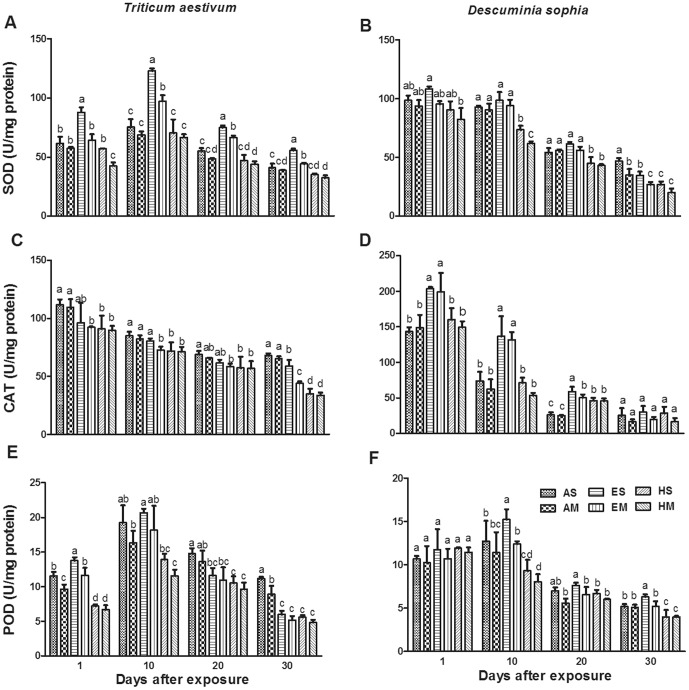
Effects of ozone on activities of superoxide dismutase, catalase and peroxidase. Activities of SOD, CAT and POD of winter wheat were shown in panel A, C and E, respectively. These of flixweed were shown in panel B, D and F, respectively. Error bar indicates SE. *n* = 9. T bars with different letters are significantly different between treatments at *P*≤0.05 at the same sampling time.

To investigate the response of antioxidant system to O_3_ and explain the physiological mechanisms by which plants accumulate different H_2_O_2_ and MDA under O_3_ fumigation, we continually monitored the dynamic patterns in the activities of SOD, CAT and POD, which are the key enzymes responsible for removing ROS induced by oxidative stress. For wheat, O_3_ significantly (*P*≤0.01) affected the activities of SOD, CAT, and POD in varying degrees during the 30 exposure days (Figure 3). As shown in Figure 3A, the SOD activity of wheat elevated at the beginning and declined afterward with the increasing [O_3_]. The activity of POD expressed a similar trend after fumigation of one day (Figure 3E). However, CAT activity was significantly depressed at each O_3_ level (*P*≤0.05, Figure 3C).

In the absence or presence of wheat, the activity of CAT in flixweed increased at elevated [O_3_], but reduced at high [O_3_] during the whole fumigating phase (Figure 3D). The activities of SOD and POD displayed a downward trend along with the time of fumigation (Figure 3B, F).

### Expression of antioxidant enzymes related genes

**Figure 4:**
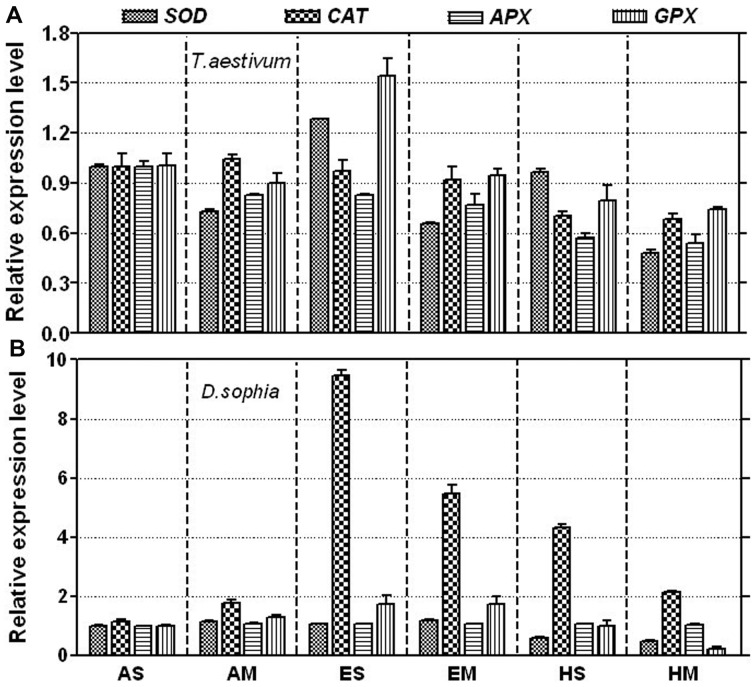
The relative expression level of antioxidant enzyme related genes . Panel A and B revealed the expression levels of these genes in wheat and flixweed leaves after fumigating for one day, respectively. Each one gene of SOD, CAT, APX and GPX was analyzed in this experiment. Data are mean±SE of three biological replicates.

In order to give an accurate explanation of the changing trend of antioxidant enzymes activities, the effect of ozone on the expression level of antioxidant enzymes related genes were examined at transcriptional levels by real-time qPCR after one day of O_3_ exposure. As shown in Figure 4A, elevated [O_3_] up-regulated the expression of SOD and GPX related genes in winter wheat. Meanwhile, competition from weed depressed them. For flixweed, O_3_ significantly up-regulated the transcript level of CAT related gene by 8.5 or 3.2 folds, respectively for ES and HS treatments. The effects of competition on the transcript level of antioxidant enzymes related genes in flixweed were similar as wheat (Figure 4B).

### Biomass and grain yield

**Figure 5:**
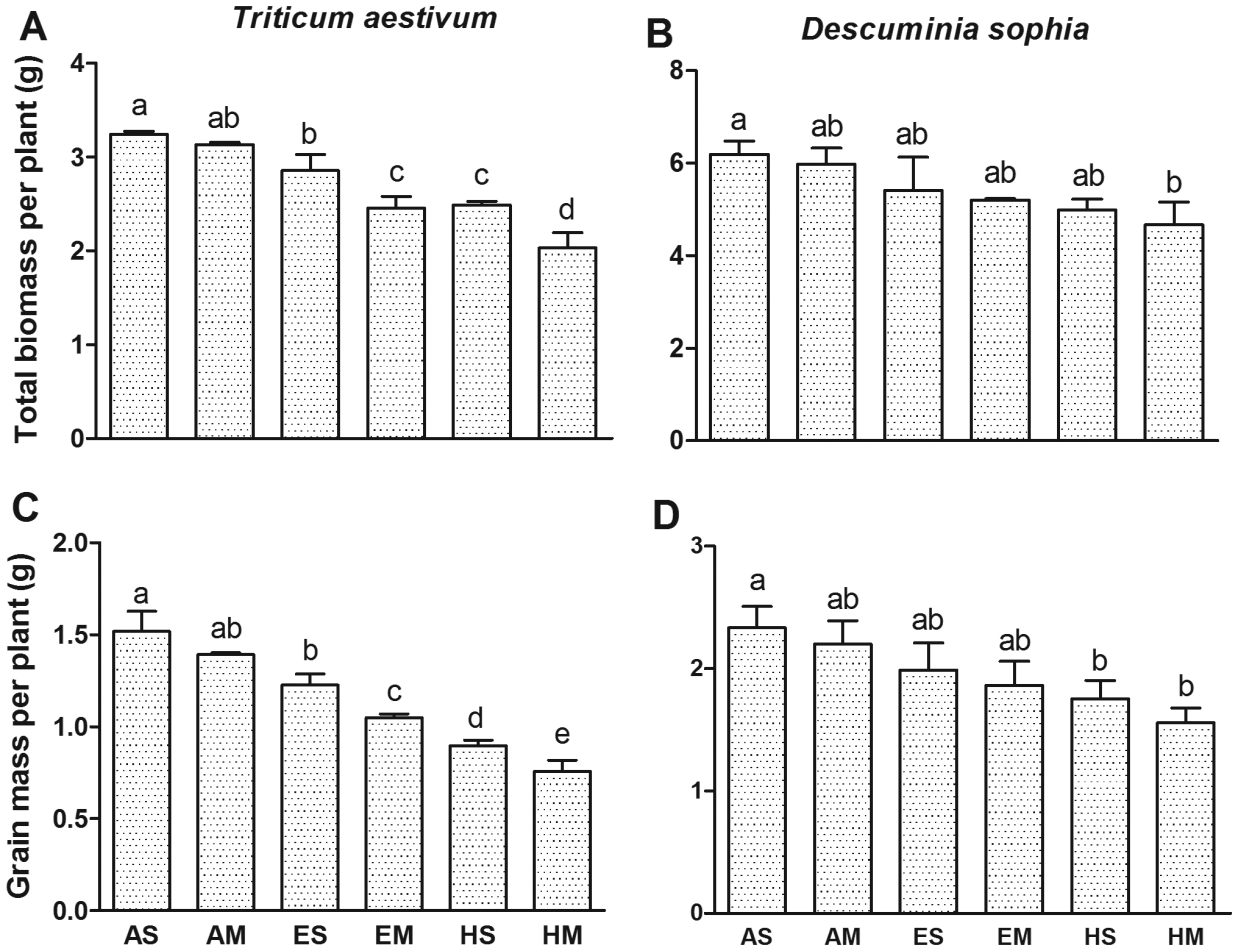
The response of biomass and grain yield in wheat and flixweed . Biomass of wheat and flixweed was shown in panel A and B, respectively. Grain mass of wheat and flixweed was shown in panel C and D, respectively. Error bar indicates SE. *n* = 15. T bars with different letters are significantly different between treatments at *P*≤0.05.

Ozone exposure significantly depressed the biomass of winter wheat by 12%, 24%, 23% and 37% respectively for ES, EM, HS and HM, against AS treatment (*P*≤0.05, Figure 5A). For wheat growing without competition from flixweed, grain yield was notably reduced by 19% and 40% at elevated (80 ppb) and high [O_3_] (120 ppb), respectively. However, for those growing with flixweed, the grain yield decreased by 31% and 50% at elevated [O_3_] and high [O_3_], respectively against AS (*P*≤0.05, Figure 5C).

In the absence or presence of wheat, the biomass and grain yield of flixweed significantly declined with the increasing [O_3_] concentration (Figure 5B, D). Against AS treatment, the biomass of flixweed was reduced by 13%, 16%, 19% and 25%, respectively for ES, EM, HS and HM. Grain yield showed similar tendency as biomass (Figure 5D).

## Discussion

### Differences in contents of chlorophyll, H_2_O_2_ and MDA under O_3_ and competition

Ozone-induced oxidative stress not only decreases chlorophyll synthesis but also decomposes the original chlorophyll in plant tissues [32]. In the present study, chlorophyll contents of both winter wheat and flixweed significantly declined under the conditions of both elevated [O_3_] and high [O_3_] (Figure 1). It is the typical symbol of O_3_ impacts on leaf pigmentation in plants as documented by previous investigations [16], [33]. However, the reduction in flixweed was less than wheat under the same treatment, suggesting flixweed was more tolerant to O_3_ than wheat. On the other hand, the effects of competition on chlorophyll content of wheat emerged at elevated and high [O_3_], while that of flixweed emerged only at high [O_3_], implying the effects of competition on wheat was more obvious than flixweed. Great reductions in chlorophyll contents are likely to decrease net photosynthetic rate, and eventually leads to biomass losses (Figure 5).

Once entering into aqueous solutions of protoplasm, ozone will decompose to reactive oxygen species like hydrogen peroxide (H_2_O_2_), singlet oxygen (O_2_^−^) and hydroxyl radicals (·OH) [34]. In addition, ozone is well known to affect the plasma function by disorganizing the membrane structure and altering membrane permeability through lipid peroxidation [35]. In the present study, H_2_O_2_ and MDA accumulation were drastically enhanced by O_3_ pollution (Figure 2), indicating that O_3_ intensified accumulation of ROS induced by oxidative stress and degree of lipid peroxidation of leaf issue membrane [14], [36]. Nevertheless, the competition aggravated the effects of oxidative stress. Although wheat or flixweed is not directly involved in producing oxidative stress, it might be correlated with the generation of ROS because of strong competition for water and nutrient resources. Even though the biochemical indicators showed similar tendency, winter wheat exhibited greater growth potential in H_2_O_2_ and MDA accumulation due to its high susceptibility to O_3_ [9].

### Differences in the antioxidant activities and expression levels of antioxidant enzymes related genes

The activities of antioxidant enzymes can be considered as a parameter of tolerance of oxidative stress. We found that O_3_ exposure caused remarkable changes activities of SOD, CAT and POD in both winter wheat and flixweed after one day exposure (Figure 3). A well-known mechanism to suppress excessive production of ROS is through the SOD which converts superoxide radicals into H_2_O_2_ and molecular oxygen. H_2_O_2_ can be further neutralized by organelle specific CAT [37]. Meanwhile, CAT metabolizes H_2_O_2_ into water and O_2_, while POD decomposes H_2_O_2_ by oxidation of co-substrates such as phenolic compounds and/or antioxidants. For winter wheat, activities of SOD and POD were stimulated in elevated [O_3_] (80 ppb), but decreased in high [O_3_] (120 ppb) after one day exposure (Figure 3A, E). These results indicated that the O_3_-induced accumulation of ROS triggered the activities of SOD and POD in wheat exposed to O_3_ at 80 ppb, which were considered to be ROS scavenging enzymes. On one hand, the production of large amounts of ROS might lead to the reduction in the activities of SOD and POD in wheat exposed to O_3_ at 120 ppb. On the other hand, the activity of CAT in winter wheat was significantly reduced by the exposure to O_3_ at either 80 or 120 ppb, which was coincident with that obtained in the previous study on two Japanese winter wheat cultivars [38]. To explore the mechanisms of antioxidant varieties under ozone pollution, we investigated the expression levels of related genes that are known to be responsible for enzyme production. According to the transcript levels of antioxidant enzymes related genes, associated with the synthesis of these enzymes, SOD and POD positively resisted the damage induced by O_3_ stress (Figure 4A). Wherefore, they are much more sensitive to O_3_ and might play more important role in eliminating ROS induced by O_3_ stress than CAT in winter wheat.

Differential exhibitions of antioxidant enzymes were obvious in flixweed, the common weed in wheat fields. Although the activities of SOD and POD were not significantly different between O_3_ and control treatment after one day exposure (Figure 3B, F), CAT was dramatically inactivated to scavenging ROS induced by O_3_ stress. The expression of CAT related gene was also significantly up-regulated in O_3_ treatments as compared with the control, which could provide explanation to the variable trend of CAT activity (Figure 4B). Furthermore, the increased expression of CAT related gene in flixweed was much higher than that of SOD and POD. This result suggested that CAT is more sensitive to oxidative stress induced by O_3_ and might play more important role in removing H_2_O_2_ for flixweed. With the increasing exposure time, antioxidant enzymes activities decreased gradually in both wheat and flixweed. This study revealed that antioxidant activities were developmentally regulated and markedly affected by O_3_ [39]. Such responses, aims at enhancing plant adaptability to environmental stress, showed a course of dynamic change under O_3_ from short-term moderate treatment to perennial severe treatment.

### Biomass and yield in response to O_3_ and competition

Ozone generates reactive oxygen species (ROS), therefore accelerates lipid peroxidation, photosynthetic pigment decomposition, and decreases CO_2_ assimilation and biomass accumulation [40]. The O_3_-induced reduction in biomass has been reported for a wide range of crop species, such as soybean, rice, cotton and wheat [14], [41]–[43]. However, little is known about how those species respond to O_3_ pollution when weed competes with crops. In this study, winter wheat (growing either singly or in mixture) displayed less dry biomass, and grain yield under O_3_ treatments against the control. Such effects may be owing to a reduction in net photosynthesis, alterations in the source-sink balance and assimilate partitioning [42], [44], [45]. We found that wheat grain yield was strongly affected by both elevated [O_3_] and high [O_3_] (Figure 5), which was similar to the results of many reports [14], [46]. Other side, the biomass and grain yield of flixweed were also reduced by O_3_ pollution (Figure 5B, D), however, they were much less than that of wheat. Ozone not only depressed the growth and development of wheat and weed, but also altered the competitive pattern between wheat and flixweed, being in favor of flixweed. The competition effects of wheat on biomass and grain yield emerged significantly under both elevated [O_3_] and high [O_3_], but that of flixweed emerged only under the high [O_3_]. Alterations of the competitive balance expressed by total biomass and grain yield were similar to Li *et al.* (1999) [22], who argued that higher antioxidant capacity and strong survivability to O_3_ were the main causes of stress adaption and species competition superiority. In many weedy species, short life cycles, as well as prolific production and dispersal, will accelerate adaptation to higher ambient O_3_ concentration [33]. From the above mentioned findings, we believe that the higher negative response of wheat is due to the competition co-existed between wheat and flixweed, and to higher susceptibility of wheat to O_3_ stress during the seed filling stage [47]. Under the normal condition, the competition between the weed and wheat was effective, and the weed was more competitive than the wheat. Moreover, under the O_3_ fumigation, the loss of wheat caused by the competition was more than that of the weed compared to the normal condition (Figure 5). Therefore, there were interaction between ozone pollution and competition, and ozone intensified the adverse effect of competition on plants.

For the past decades, yield of wheat cultivars has been improved by a great degree at the expenses of their resistances to environmental stress [14], [43]. Consequently, other factors were overlooked in the process of crop breeding, such as O_3_ pollution, competition from weeds and so on. Actually, competition has been demonstrated to be an important factor affecting plant responses to O_3_ stress [23]. In the agro-ecosystem, wheat production is not only confronted with O_3_ pollution but also competition from weed, which has exhibited moderate to high levels of tribenuron resistance [17]. Therefore, adversity resistance should be fully considered during wheat breeding as well as high yield and quality.

In summary, adverse effects of O_3_ pollution on winter wheat and weed (flixweed) have been clearly found under both competition and non-competition conditions. And these effects differed from winter wheat and flixweed, which may also cause long term changes in competition pattern by disadvantaging winter wheat more than weed. Flixweed has stronger resistance to O_3_ and is more competitive than winter wheat due to its stronger adaptability to environmental stresses. More effective weed management strategies and wheat breeding with adversity resistance should be developed in order to maintain ideal wheat production.

## Supporting Information

Figure S1 The mean daily temperature and mean monthly precipitation of 2011 at the experimental site.
(TIF)
