# Supplementary figures and images for: Flixweed Is More Competitive than Winter Wheat under Ozone Pollution: Evidences from Membrane Lipid Peroxidation, Antioxidant Enzymes and Biomass
Source: PLoS One. 2013 Mar 22;8(3):e60109. doi: 10.1371/journal.pone.0060109 (PMC3606213; doi:10.1371/journal.pone.0060109)

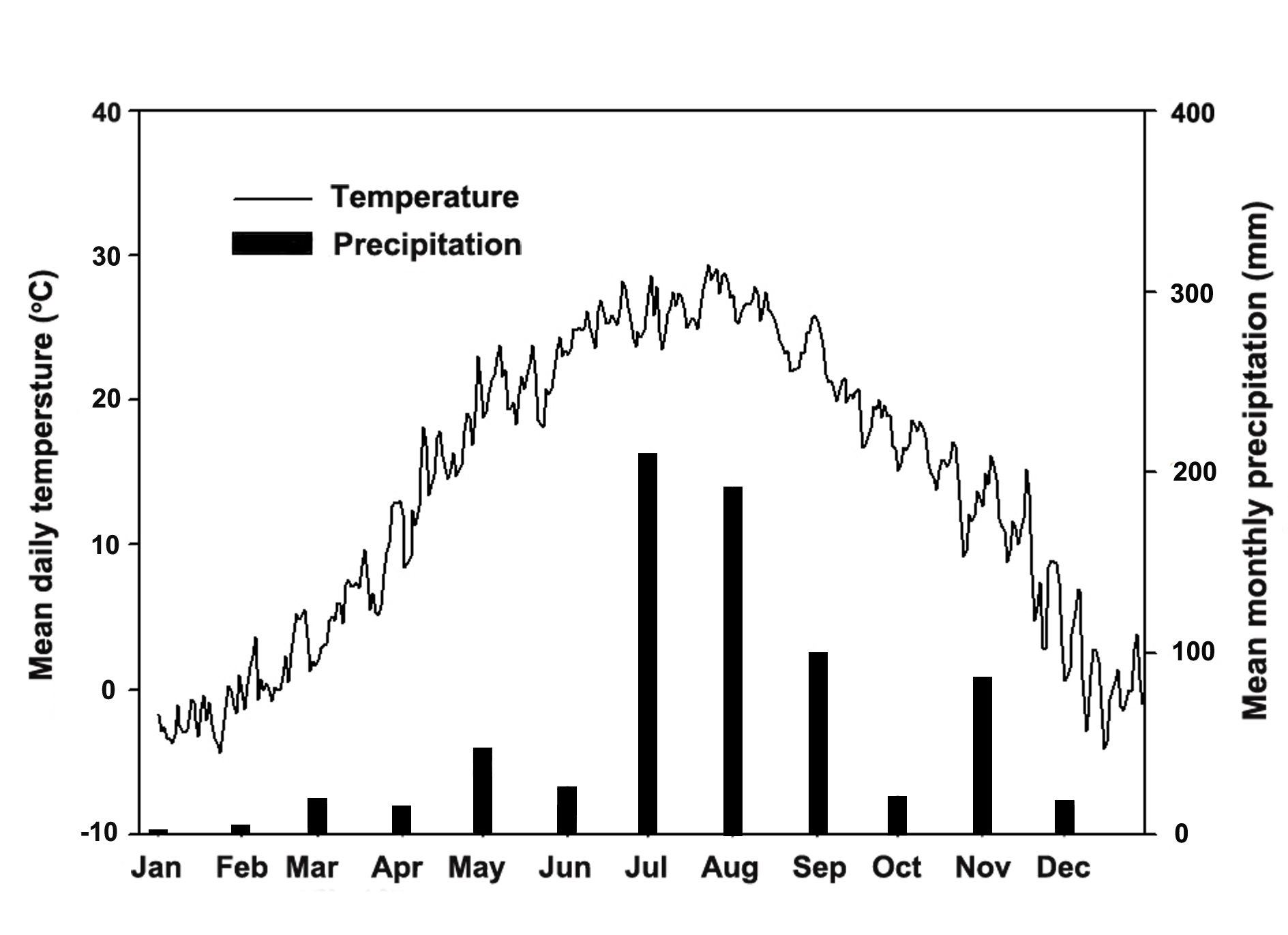

Supplement: Figure S1 — The mean daily temperature and mean monthly precipitation of 2011 at the experimental site. (TIF) [file pone.0060109.s001.tif]
